# Lung cancer resection rate and outcomes during the COVID-19 pandemic in Northern Finland

**DOI:** 10.2340/1651-226X.2025.44535

**Published:** 2025-10-01

**Authors:** Aapo Pikkujämsä, Tuomas Mäkelä, Olli Helminen, Joonas H. Kauppila, Fredrik Yannopoulos

**Affiliations:** a Translational Medicine Research Unit, Medical Research Center, University of Oulu, Oulu, Finland; b Department of Surgery, Vaasa Central Hospital, Wellbeing Services County of Ostrobothnia, Vaasa, Finland; c Department of Cardiothoracic Surgery, Oulu University Hospital, Oulu, Finland; d Department of Molecular Medicine and Surgery, Karolinska Institutet and Karolinska University Hospital, Stockholm, Sweden

## Introduction

Lung cancer is the second most diagnosed cancer worldwide and the leading cause of cancer-related mortality [1]. In Finland, over 2,800 new lung cancer cases are diagnosed each year [2]. Surgery is the primary treatment in early-stage non-small cell lung cancer (NSCLC), and in Finland, approximately 15% of all patients undergo surgical resection. In Stage I disease, the resection rate can be as high as 50% [3, 4]. Lung cancer survival has improved across the Nordic countries in recent decades. Norway has the highest overall 5-year lung cancer survival rate, at 26.6% for men and 33.2% for women. In contrast, Finland has the lowest survival rates, with 16.4% for men and 25.5% for women [5]. The reasons for these discrepancies in mortality remain unclear, despite the relatively comparable healthcare systems in the Nordic region. Early detection and a higher resection rate may be a key factor in survival differences, as Norway’s reported resection rate ranges from 18.1 to 22.5%, which is considerably higher than that of Finland [6].

The COVID-19 pandemic in 2020 and 2021 led to widespread lockdowns and placed excessive burden on healthcare globally. As healthcare resources were redirected towards treating COVID-19 patients, concerns arose regarding the potential impact on the treatment of non-COVID-related diseases, including malignancies. Studies have reported a decline in colorectal cancer diagnoses in Italy during the COVID-19 pandemic years, with similar trends observed in multiple other cancer types worldwide [7, 8].

We hypothesised that lung cancer resection rates may have decreased during the COVID-19 pandemic. To examine the hypothesis, we analysed lung cancer resection rates in Northern Finland from 2015 to 2022.

## Patients/material and methods

This was a retrospective, observational, single tertiary centre registry study, based on national cancer registries [2]. Finnish national registries include all newly diagnosed cancer cases from all healthcare districts. Oulu University Hospital is the tertiary care centre responsible for lung cancer surgery in all of Northern Finland. We retrospectively collected data between January 2015 and December 2022. Patients treated in 2020 and 2021 were categorised as the pandemic group. All surgically treated NSCLC patients were included in the study. Lung cancer incidence data were obtained from the Finnish Cancer Registry. Patients referred from two hospitals were excluded, as these centres performed a limited amount of operative treatment for lung cancer during the study period.

The main outcome measure was the resection rate, calculated as the ratio of surgically treated cases to diagnosed lung cancer cases.

IBM SPSS statistics software version 29.0.1.0 was used for statistical analysis.

## Results

The final analysis included 300 patients. During the study period, the total prevalence of lung cancer in the study population area was 2,000 cases. The overall resection rate was 15.0%. Patients were categorised into two groups based on whether they underwent surgery during the COVID-19 pandemic restriction years (pandemic group, *n* = 82) or outside this period (non- pandemic group, *n* = 218).

**Figure 1:**
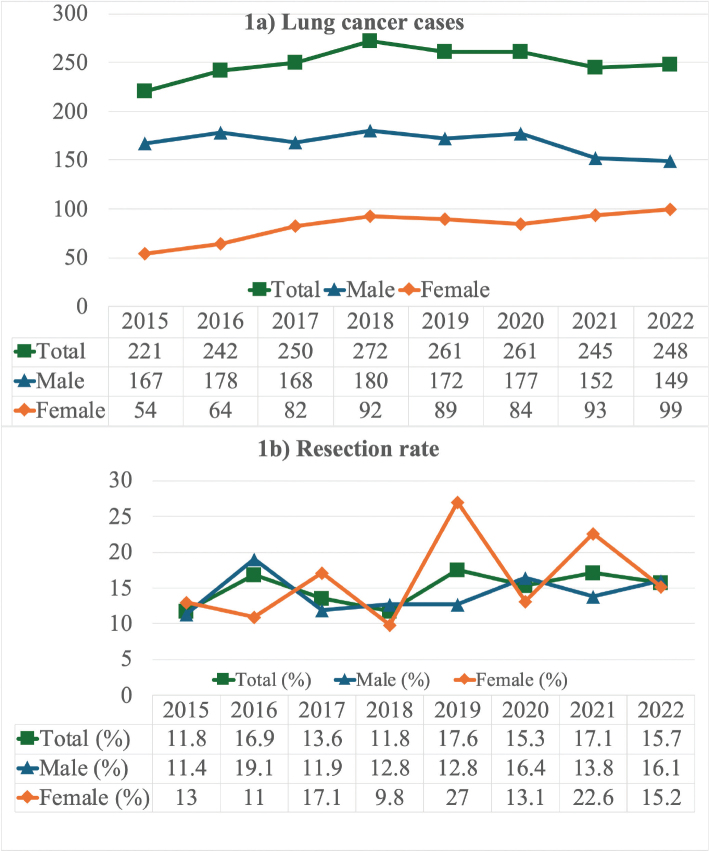
(a) Changing patterns in lung cancer incidence among men and women in Northern Finland between 2015 and 2022. (b) Annual lung cancer resection rates by gender in Northern Finland, highlighting evolving trends over time from 2015 to 2022.

The mean resection rate during the COVID-19 pandemic years was 16.2 and 14.6% in non-pandemic years. There was no statistical difference in resection rate between the groups (*p* = 0.379, Figure 1). Resection rates in male patients during pandemic versus non-pandemic years were 15.2 and 14.0%, respectively (*p* = 0.589); female resection rates observed were 18.1 versus 15.8%, respectively (*p* = 0.490).

The pandemic did not have an impact on waiting times from decision to surgery: 21 and 22 days respectively (*p* = 0.947). Within the pandemic group, average first-second forced expiratory volume (FEV1) was 87.5% of the reference value, which was statistically higher (*p* < 0.001) compared to the non-pandemic group (79.4%). There was a reduction in the percentage of current or former smokers in the pandemic cohort, standing at 72.0% versus 84.4% in the non-pandemic cohort (*p* = 0.018). Overall patient demographics were similar. The proportion of final histopathological groups was similar between the two groups. The median hospital stay was 5 days in both groups (*p* = 0.162, Supplementary Table 1).

There was no difference in overall 1-year survival: 91.5% for the pandemic group and 93.1% for the non-pandemic group (*p* = 0.615 in Kaplan-Meier analysis). In the Cox regression model, there was also no statistical difference (odds ratio for pandemic group 1.26, 95% confidence interval 0.5-3.1, *p* = 0.621).

## Discussion and conclusion

The COVID-19 pandemic did not affect the rates of NSCLC resection in Northern Finland. This outcome stands out as a notable deviation when compared to other cancer types reported in various studies [8–10]. It could be speculated that the COVID-19 pandemic led to a heightened focus on evaluating respiratory symptoms, resulting in more timely diagnoses of lung cancer. More effective evaluation of respiratory symptoms may have reduced the number of missed or late diagnosis compared to other cancer types, which may have protected lung cancer resection rates from negative pandemic effects. A greater global attention to respiratory symptoms during the pandemic may partly explain the improved FEV1 values and the reduced prevalence of smoking.

This study was a retrospective cohort study, which inherently introduces limitations. Although our study is single-centre, it still represents the sole provider of surgical treatment in Northern Finland for NSCLC. This does confine the findings to Northern Finland rather than representing the entire nation. In Finland, NSCLC resection rate is lower than several European countries, such as Norway, Iceland and the Netherlands, where resection rates vary from 18.1 to 26.4% [6, 11, 12]. The lower resection rate correlates with poorer long-term patient outcomes [13]. We hypothesise that lung cancer may have been diagnosed at a non-operable stage, patients may have been deemed unfit for surgery, or the criteria for surgery are too restrictive. In the current study, Stage I percentage was lower than reported in other studies; stage I cancer accounted for 56–62% in our study, compared to 65–81% in other studies [14, 15]. This may indicate a later time of diagnosis and a more advanced stages compared to other countries.

Having lower resection rates may be the underlying reason for worse outcomes in Finland compared to other Nordic countries. It has been previously estimated that 20–25% of lung cancer patients might be operable at the time of diagnosis [16]; compared to this, our national resection rates were far from optimal. Our study shows that our resection rate needs evaluation on the national level to determine the possible explanations for the gap between Finland and other Nordic countries. Enhancing diagnostic pathways and shortening time delays might improve the resection rate.

## Supplementary Material



## Data Availability

Currently the Finnish legislation prevents full raw data sharing for the public (Act on medical research 488/1999, Data protection act 1050/2018, Act on the secondary use of health and social data 552/2019) and therefore we are unable to share our full data.
