# Supplementary material for: Lung cancer resection rate and outcomes during the COVID-19 pandemic in Northern Finland
Source: Acta Oncol. 2025 Oct 1;64:44535. doi: 10.2340/1651-226X.2025.44535 (PMC12504990; doi:10.2340/1651-226X.2025.44535)
Supplement: Supplementary file 1 [file AO-64-44535-s1.pdf]

Supplementary material has been published as submitted. It has not been copyedited, or typeset by Acta Oncologica

Supplementary Table 1. Patient demographics and outcomes

**Variable**	**Pandemic group** (n=82)	**Non-pandemic group** (n=218)	**p-value**
Age	69.2± 9.1	68.2±9.9	0.385
**BMI**	26.2±4.4	26.7±4.8	0.414
**Gender**			0.503
Female	32 (39.0%)	76 (34.9%)	
Male	50 (61.0%)	142 (65.1%)	
**CCI**			0.791
0	25 (30.5%)	56 (25.7%)	
1	26 (31.7%)	78 (35.8%)	
2	18 (30.0%)	53 (24.3%)	
≥3	13 (15.9%)	31 (14.2%)	
**DLCO** (SD)	79.8±19.3	79.6±18.8	0.92
**Waiting time** (IQR)	21 (14-33)	22 (14-29)	0.947
**Surgical technique**			0.579
RATS	10 (12.2%)	26 (11.9%)	
VATS	34 (41.5%)	77 (35.3%)	
Thoracotomy	38 (46.3%)	115 (52.8%)	
**Resection**			0.003
Pneumonectomy	1 (1.2%)	2 (0,9%)	
Sublobar	14 (17.1%)	11 (5.0%)	
Lobar	67 (81.7%)	205 (94.0%)	
**Stage**			0.439
I	51 (62.2%)	122 (56.0%)	
II	15 (18.3%)	56 (25.7%)	
III	15 (18.3%)	35 (16.1%)	
IV	0 (0.0%)	2 (0.9%)	
Undefined	1 (1.2%)	3 (1.4%)	
**Histology**			0.288

SCC	21 (25.6%)	53 (56.0%)	
Adenocarcinoma	53 (64.6%)	127 (58.3%)	
Carcinoid	4 (4.9%)	14 (6.4%)	
Other	4 (4.9%)	24 (11.0%)	
**Length of stay** (IQR)	5 (3-7)	5 (4-7)	0.162
**30-d mortality**	0 (0%)	0 (0%)	N/A
**90-d mortality**	1 (1.2%)	1 (0.5%)	0.490

CCI: Modified Charlson comorbidity index, FEV1: first second forced expiratory volume, DLCO: diffusing capacity, Waiting time: days from referral to surgery, RATS: robotic assisted thoracoscopy, VATS: video assisted thoracoscopy, SCC: Squamous cell carcinoma, Stage: pathological stage by TNM8 classification[17]
